# Freshwater wild biota exposure to microplastics: A global perspective

**DOI:** 10.1002/ece3.7844

**Published:** 2021-07-09

**Authors:** Alessandra Cera, Massimiliano Scalici

**Affiliations:** ^1^ Department of Sciences University of Roma Tre Roma Italy

**Keywords:** biodiversity, biofilm, field research, food web, inland waters, plastic

## Abstract

1. Current understanding on the exposure of freshwater organisms to microplastics (plastics sized between 1 µm and 5 mm) has arisen mostly from laboratory experiments—often conducted under artificial circumstances and with unrealistic concentrations. In order to improve scientific links through real ecosystem exposure, we review field data on the exposure of free‐living organisms to microplastics.
2. We highlight that the main outputs provided by field research are an assessment of the occurrence and, at times, the quantification of microplastics in different animal taxa. Topics of investigation also include the causes of contamination and the development of biological monitoring tools. With regard to taxa, fish, mollusks, and arthropods are at the center of the research, but birds and amphibians are also investigated. The ingestion or occurrence of microplastics in organs and tissues, such as livers and muscles, are the main data obtained. Microorganisms are studied differently than other taxa, highlighting interesting aspects on the freshwater plastisphere, for example, related to the structure and functionality of communities. Many taxa, that is, mammals, reptiles, and plants, are still under‐examined with regard to exposure to microplastics; this is surprising as they are generally endangered.
3. As biota contamination is acknowledged, we contribute to an interdisciplinary scientific discussion aimed at a better assessment of knowledge gaps on methodology, impact assessment, and monitoring.

## Graphical abstract

Research on microplastics in freshwater biota is increasing in recent years. Several taxa are investigated, most of all fish. Methodology, impact assessment and biomonitoring are issues to be further investigated.

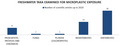

## 1 INTRODUCTION

Plastics are organic synthetic polymers that have registered steady growth since the beginning of their production in the 20th century. Indeed, global production exceeded 380×10^9^ kg in 2019 (PlasticsEurope, 2020). Mismanaged plastic waste has become a growing global issue as it causes extensive soil and water contamination (Lacerda et al., 2019; Windsor et al., 2019). Plastics are a persistent pollutant, as they have a low rate of degradation: Their degradation rate is lower than their contamination rate, leading to an accumulation of mismanaged plastic waste in the environment. When plastics are exposed to environmental conditions, physical, chemical, and biological agents can break them into smaller particles by photo‐oxidation, thermal, and mechanical degradation (Singh & Sharma, 2008).

Microplastics (MPs) are plastics of size between 1 µm and 5 mm (Frias & Nesh, 2019; Gilgault et al., 2018; Thompson et al., 2004). They can be called primary MPs, if directly produced by industrial activities (such as the cosmetic industry), or secondary MPs, if generated by the degradation of larger plastics due to environmental factors such as those described above. MPs contaminate air, soil, and water, including ice, worldwide (Guo et al., 2020; Petersen & Hubbart, 2021; Zhang et al., 2020).

Freshwater MPs are found in rivers and lakes on all continents, including a recent discovery in Antarctica (González‐Pleiter et al., 2020). MP contamination is not uniformly distributed around the globe and varies greatly on a local scale. Southeast Asia and Europe generally have a higher concentration of MPs in inland waters; however, some areas show knowledge gaps in sampling, especially Africa and Oceania. There is also a difference between freshwater types as lakes are generally more contaminated than rivers. Furthermore, both riverine and lacustrine sediments are proposed, given that MPs sink as they are more contaminated than the aquatic matrix (see Cera et al., 2020). The main MP contaminants are polypropylene (PP) and polyethylene (PE), followed by polystyrene (PS). These polymers are the prevalent MP contaminants in water and sediments, while PP and PE are also mainly detected in fish and macroinvertebrates (see Cera et al., 2020). MP origins are diverse and include atmospheric deposition, water runoff, and inefficient wastewater treatment plants (Free et al., 2014).

Research on MPs and biota has mainly focused on marine rather than freshwater organisms (Blettler et al., 2018), and their effects are partially understood, although MPs are to date considered a threat to freshwater ecosystems (Li, Busquets, et al., 2020; Reid et al., 2019). In fact, MPs (also in combination with other contaminants) negatively impact some processes in animal organisms at molecular, cellular, and individual level, for instance on stress response genes, intestinal epithelium, and gametogenesis (Xu et al., 2020). Nevertheless, a few studies have assessed some impacts on freshwater species. For instance, 5–20 µm PS beads induce toxicity in the liver of *Danio* *rerio* at concentrations of 10^3^ and 10^5^ particles L^−1^ (Lu et al., 2016) which is over the global mean value found in inland waters (see Cera et al., 2020). PE alters gene transcription for 14 days when developing *D*. *rerio* larvae are exposed to a concentration of about 480 particles L^−1^ (LeMoine et al., 2018), which is a realistic concentration found in nature. PE also impacts the microbiome of *D*. *rerio* larvae (Wan et al., 2019). As for adults, different MP polymers, such as PE, polyamides, PP, and polyvinyl chloride, damage the intestine of adults (Lei et al., 2018). Regarding other species, *Daphnia magna* immobilizes when it ingests 1–4 µm PE spheres (Rehse et al., 2016). It is to be considered that MPs do not always cause negative effects on organisms. For instance, the same experiment on *D*. *magna* conducted by Rehse et al. (2016) did not produce an effect for larger MPs, that is, 90–106 µm. In addition, high MP uptake does not necessarily cause high toxicity, as demonstrated in *Dreissena polymorpha* when exposed to a mixture of 1–10 PS µm microbeads at the concentration of 10^5^ and 10^6^ particles L^−1^ (Magni et al., 2018). Some microorganisms can also benefit from the exposure to MPs because they can degrade MPs and use them for cell growth (Taipale et al., 2019).

Microplastic studies are mainly conducted in the laboratory and few studies evaluate their impacts in the natural field (O’Connor et al., 2019). Moreover, the collection and analysis of MPs from freshwater field data are currently conducted globally by diverse and not standardized methods (O’Connor et al., 2019). This could affect the comparison of results.

As recent available reviews on MPs focus on marine biota (Rezania et al., 2018), laboratory studies (Li, Busquets, et al., 2020; Li, Su, et al., 2020), or methodology (O’Connor et al., 2019), our main purpose is to focus on the information obtained from field observations on freshwater biota, providing an overview by taxa. We collect and discuss recent scientific literature reporting data from inland waters where organisms have been exposed to MPs, where by MP exposure we mean any process potentially affecting living organisms, for instance, their alimentary strategy and growth. Finally, this review provides a future perspective for (a) highlighting knowledge gaps, and (b) evaluating the potential of biota as an MP monitoring tool for the health of ecosystems.

## 2 METHODS

Bibliographic research of peer‐reviewed international articles was conducted on Scopus and ISI Web of Knowledge with the following keywords: “microplastics” AND “rivers”; “microplastics” AND “lakes”; “microplastics” AND “freshwater” AND “plants” or “phytoplankton” or “algae”. Subsequently, a refined search was conducted with the word “biota”. A first manual selection of articles of interest was based on the available information in the title and abstract. A second round selected the final articles based on a thorough content check. Studies on biota in lentic and lotic ecosystems were selected up to 20 July 2020. There were no lower time limits. The prevalence of studies by ecosystem and year of publication was represented graphically. The type of biota investigated was recorded for each ecosystem according to the taxa. The results were graphed and discussed first globally, then specifically by taxonomic group. Summary tables are provided for invertebrates, with the exception of Annelida (because only one species was analyzed), and vertebrates. The information in the tables is collected from the text and on occasion from the graphs of the articles obtained from the bibliographic search. As the results are collected by species, the results of more than one species pooled together are not considered. Benthic invertebrates can be indicated at the taxonomic level of order or family as they can be difficult to identify at specific level.

## 3 RESULTS

**FIGURE 1:**
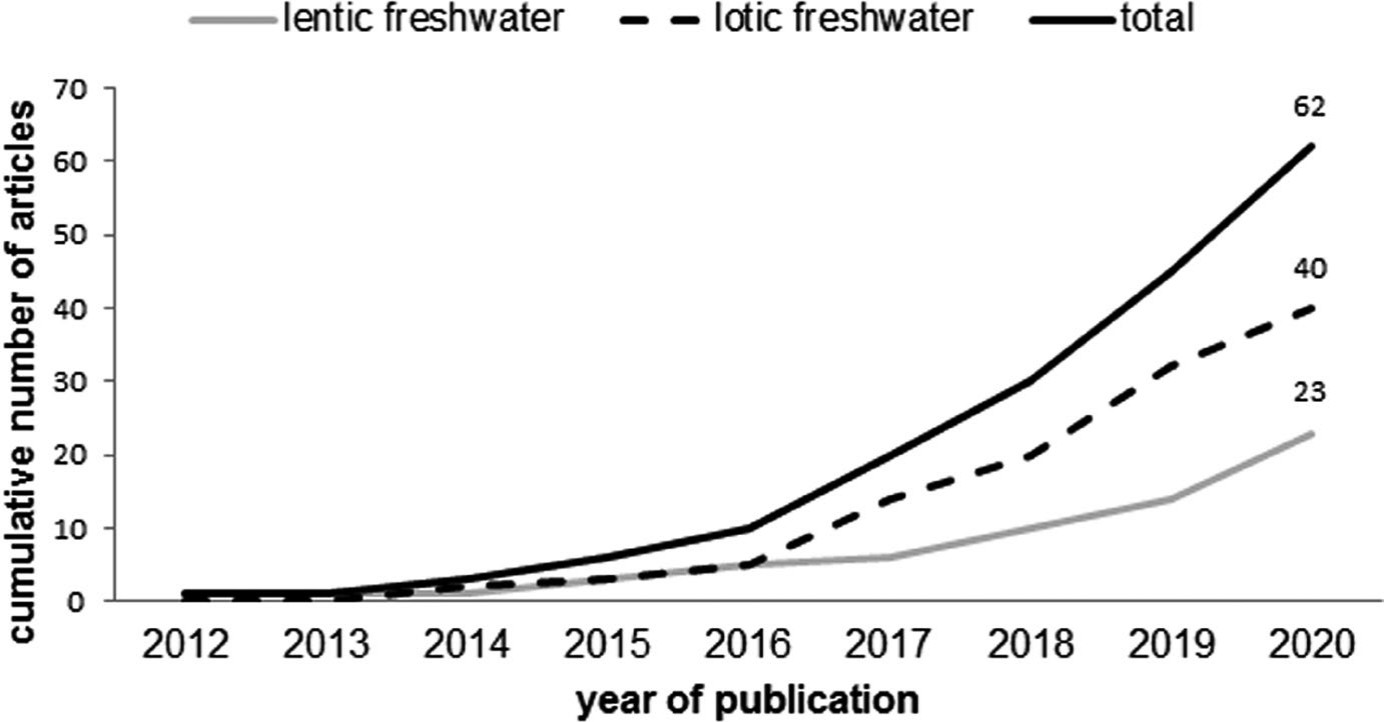
Publication trends of scientific articles on microplastics in freshwater wild biota in total and by ecosystem type (lentic, lotic). One article does not provide a specific ecosystem type and is thus included only in the total count; two articles examine both lentic and lotic fresh waters and are included in both ecosystem types

An in‐depth literature search collected 62 scientific articles from 2012 to 2020 (Appendix S1). The number of publications has increased in recent years, by total number and by type of aquatic habitat, that is, lentic or lotic (Figure 1). The first evident finding is the rapid increase in publication in the last decade, while the second regards the exposure of biota to MPs in lentic ecosystems, the latter being less investigated than lotic ecosystems (Figure 1).

**FIGURE 2:**
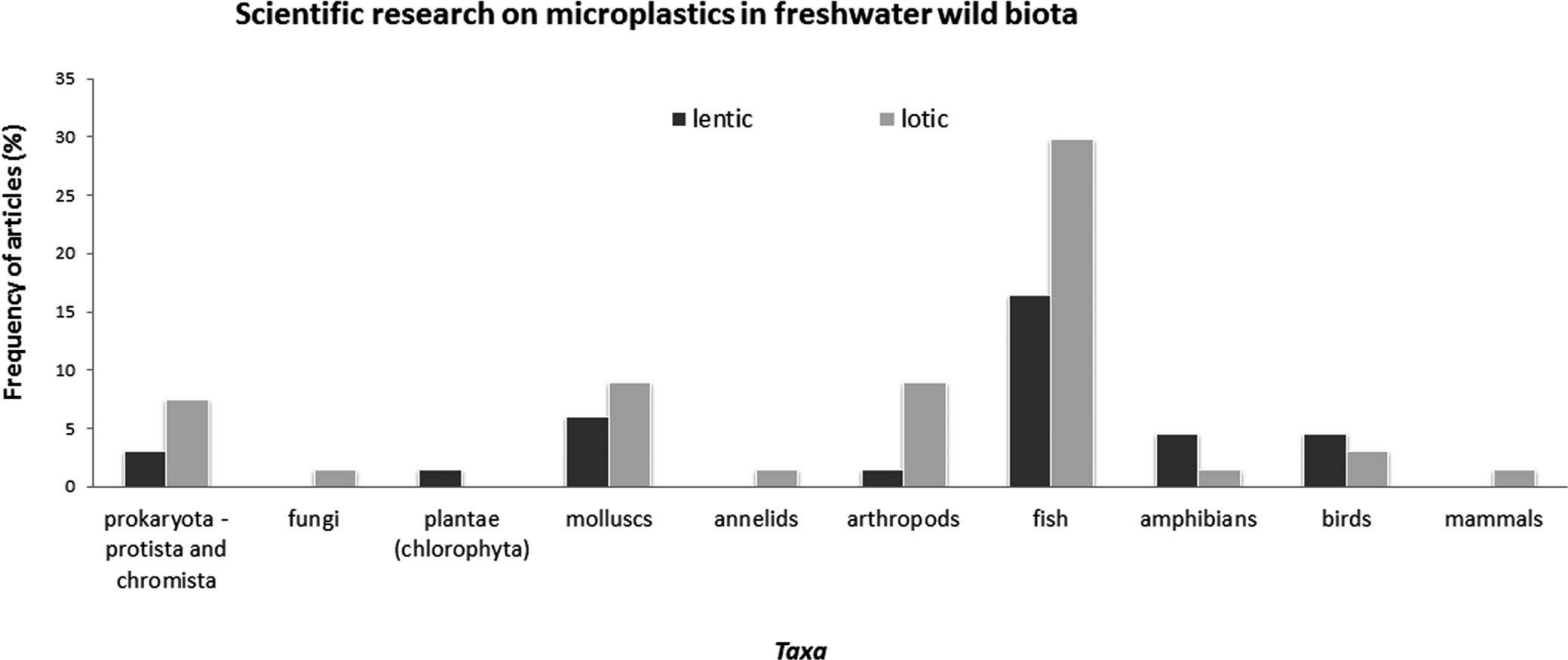
The occurrence of scientific literature on MP in fresh waters (lentic or lotic) according to taxa: Prokaryota, Protista and Chromista, Fungi, Plantae (Chlorophyta), invertebrates (mollusks, annelids, arthropods), and vertebrates (fish, amphibians, birds)

Microorganisms, including bacteria, cyanobacteria, algae, and fungi, are studied to determine colonization on MPs, especially in lotic ecosystems (Figure 2). Microorganisms are studied in 14% of the articles (*n* = 9). Current studies on microorganisms do not focus on the harmful impacts on organisms as studies do on other taxa, but rather on the implications for the structure and functionality of communities.

Invertebrates are studied more in lotic than in lentic ecosystems (Figure 2). Among invertebrates, Mollusca is the main taxonomic group investigated (15% of total studies, *n* = 10). Bivalves are the main taxa studied in lotic fresh waters, and they are the only taxa studied in lentic fresh waters. Apart from Mollusca, seven studies (10% of the total studies) examine Arthropoda (Insecta and Crustacea), while one study is on Annelida (1% of total studies).

Freshwater vertebrates are investigated more often than microorganisms and invertebrates. In particular, fish are the preferred research model for MPs in both lentic and lotic ecosystems, as 45% of studies focus on fish (*n* = 30) (Figure 2). Birds accounted for 9% of the total studies (*n* = 6). Birds are sampled mainly in lentic ecosystems, except for the study of Holland et al. (2016), which samples water birds from a large geographical area, which may include rivers. Only four studies concern amphibians (6% of the total studies), and no study concerns reptiles or mammals (Figure 2). The following paragraphs describe the results of the scientific literature in detail by taxa.

### 3.1 Prokaryota and Eukaryota (Protista, Chromista)

Marine studies proved that buoyant plastic can be colonized by microorganisms, commonly known as “plastisphere” (Zettler et al., 2013). Inland ecosystems are subject to the same phenomena.

Natural and anthropogenic factors influence the plastisphere. In lakes, the microbial communities that colonize MPs were compared with those living in the surrounding waters in three types of lakes: oligo‐mesotrophic, eutrophic, and dystrophic. Microbial colonization was abundant in oligo‐mesotrophic and dystrophic lakes, consuming significantly more oxygen than in eutrophic lakes. Functional diversity differed from surrounding aquatic communities for all three lake types, highlighting an impact due to MPs. A significant difference was also detected in functional richness between the community on MPs and in water. These results suggest an environmental impact on heterotrophic activities and possibly on the carbon cycle in lakes (Arias‐Andres et al., 2018). In addition, the depth of the buoyant plastics and the type of polymer also affect the plastisphere, in particular bacteria, cyanobacteria, and algae (Leiser et al., 2020).

Regarding anthropogenic activities, the effects of effluents from wastewater treatment plants were investigated by microbial assemblages on the MPs in rivers. Two main aspects have been demonstrated: Eukaryotic and prokaryotic assemblages on MPs are significantly different from those in water, seston, and benthos (Hoellein et al., 2017; Kettner et al., 2019; McCormick et al., 2014; Oberbeckmann et al., 2018); assemblages downstream and closer to wastewater treatment plants provide more species related to human sources, such as gastrointestinal species (e.g., *Arcobacter*), organisms that can decompose plastics (e.g., *Pseudomonas*), species potentially harmful to the environment (e.g., *Pfiesteria*), and species linked to antibiotic resistance, suggesting MPs as a horizontal gene transfer hotspot (Hoellein et al., 2017; Kettner et al., 2019; McCormick et al., 2016; Oberbeckmann et al., 2018).

Regarding eukaryotes, among the 500 taxa found on MPs, animal DNA belonging to Annelida, Rotifera, and Nematoda was also found. Larval or juvenile stages are assumed to live on buoyant MPs, although there is a possibility that environmental DNA has been detected (Kettner et al., 2019).

### 3.2 Fungi

The colonization of fungi on MPs has not been thoroughly studied. A single scientific article evidences the occurrence of MP colonization by fungi, which represents 22% of the 793 total eukaryotic taxa. The presence of Chytridiomycota, Cryptomycota, and Ascomycota was assessed (Kettner et al., 2017).

### 3.3 Plantae

#### 3.3.1 Chlorophyta

The impacts of MPs on plants in nature are found in one study. The colonization of MPs by Chlorophyta and other microorganisms, such as Cyanophyta and algae (Bacillariophyta, Cyanophyta, Cryptophyta, Euglenophyta, Pyrophyta), has been studied, with the finding that colonization varies according to the season, that is, MPs are colonized more during the summer than in other seasons (Chen et al., 2019). Since MP buoyancy is affected by the weight of the colony of microorganisms, MPs have been observed to be less buoyant during the summer (Chen et al., 2019). It is suggested that the effects of microorganisms on the buoyancy of MPs during remediation activities can be considered. For instance, cleaning the surface of water bodies could be more effective during winter, when MP buoyancy is less affected by colonization. In addition, further evaluation of the algal species colonizing MPs is suggested in order to investigate the potential effect of MP contamination on algal blooms.

#### 3.3.2 Viridiplantae

Our bibliographic research found no scientific articles on viridiplantae and MP observations in field studies. Laboratory experiments showed that MPs appear to have limited effects on vascular aquatic plants (Dovidat et al., 2019; Mateos‐Cárdenas et al., 2019); for instance, MPs inhibit the growth of shoots and roots (Kalčíková et al., 2017; Pflugmacher et al., 2020). In addition, MPs are adsorbed and accumulated, showing potential depuration activity but at the same time they can be a site of entry into the food web due to the feeding activity of animals (Kalčíková, 2020). Since vascular aquatic plants are currently under‐studied, but laboratory experiments have proven some impacts due to MPs, it is suggested that the development of a method for information gathering be considered. For instance, plants could be exploited to assess chronic contamination of a specific site.

### 3.4 Invertebrates

#### 3.4.1 Mollusca

Mollusca is the main invertebrate Phylum investigated (Appendix S2). However, only a few collected studies analyze gastropods (e.g., Xu et al., 2019), while most focus on bivalves. Starting from the interactions of MPs on gastropods, two species, namely *Lanistes varicus* (Müller, 1774) and *Melanoides tuberculata* (Müller, 1774), collected from the Osun River system (Nigeria), were compared with *Theodoxus fluviatilis* (Linnaeus, 1758), collected from the Rhine River (Germany). All species contained MPs and fibers were the dominant particle shape in all three species, although they belong to different sampling areas. The concentrations of MPs per individual differ statistically, as follows: *L*. *varicus* > *M*. *tuberculate* >> *T*. *fluviatilis*. This result is explained by Akindele et al. (2019) by the fact that larger gastropods (in this case *L*. *varicus* and *M*. *tuberculata*) require more nutrients and therefore ingest more MPs during food consumption than smaller gastropods (such as *T*. *fluviatilis*). However, the trend is reversed if the concentration is measured by the number of MPs per wet weight: *T*. *fluviatilis* shows a significantly higher concentration of MPs than *M*. *tuberculata* and *L*. *varicus*. As highlighted by this study, the unit of measurement plays a fundamental role in the evaluation of results and in the classification of potential risk for organisms (see section 4 “Knowledge gaps”).

Regarding bivalves in rivers, *Anodonta anatina* L., 1758 has been shown to accumulate more MPs downstream of an urban area with two wastewater treatment plants than a rural one, thus suggesting an impact induced by the greater density of human population (Berglund et al., 2019). The type of MP found is mainly fiber, although spherules also occur (Berglund et al., 2019). Another species, *Unio pictorum* L., 1758 was sampled upstream, downstream, and at the effluent of a sewage treatment plant, showing accumulation of MPs only in the latter site but with low values (Domogalla‐Urbansky et al., 2019). Lastly, small (<3 cm) Dreissenids bivalves, that is, *D. polymorpha* (Pallas, 1771) and *Dreissena bugensis* (Andrusov, 1897), were sampled to evaluate the accumulation of MPs. MP microbeads were detected in sediment samples; however, they were absent in the 147 collected specimens, suggesting that the animals were too small to filter out MPs >35 µm (Schessl et al., 2019). Comparing these results, it is suggested that only large (>3 cm) bivalve species are capable of accumulating MP microbeads present in the environment. However, as the sampling of MPs in water and sediments is not performed by Berglund et al. (2019) or Domogalla‐Urbansky et al. (2019), it is not possible to make a comparison to evaluate the concentration detected inside animals and that of the environment. Nevertheless, sewage and wastewater treatment plants are suggested as being an input source of MPs into fresh waters and biota contamination.

#### 3.4.2 Annelida

To date, one study has analyzed the interactions between Annelida and MP. In detail, 48% of *Tubifex tubifex* (Müller, 1774) ingested MPs with a mean of 129 ± 65.4 particles per gram of tissue with no obvious effects on their fitness. Fibers were the most abundant MP shape, while polyester, polyethylene terephthalate, and polystyrene were the main types of polymers. Interestingly, the microbeads were not detected in the worms even though they were present in the surrounding sediment, suggesting a possible selectivity induced by particle size. Only particles of MPs <63 µm are ingested by *T*. *tubifex*, while microbeads between 124 and 1,050 µm are excluded. This selectivity could cause some MPs, such as fibers, to be transferred more easily through the trophic web. Transfer is also enhanced by the observation that MPs showed a longer residence time than nonplastic material in the gut (Hurley et al., 2017). The results obtained from this first observation are not comparable, to our knowledge, with other scientific articles on this topic; however, a new research scenario has been opened, especially considering the role of Annelida in the food web.

#### 3.4.3 Arthropoda

Among arthropods, mainly insects were analyzed, although some studies concern crustaceans (e.g., Nan et al., 2020) (Appendix S3). 50% of Baetidae, Heptageniidae (both belonging to Ephemeroptera), and Hydropsychidae (Trichoptera) contained MPs from sampling sites near highly urbanized areas (South Wales, UK). After gut evacuation, nearly half of the MPs were expelled, showing a significant reduction. The pre‐evacuation concentration of MPs varied between families; however, it was not related to feeding guild, that is, filter‐feeders or detritivores. The variation has been explained to a limited extent by the characteristics of the habitat, such as the volume of the river flow—if the volume is greater, MPs decrease—and by the wastewater treatment plant—if the effluent discharge is greater, MPs increase. Indeed, it is suggested that where the contamination is minor, dilution by adding water improves the reduction in MP concentration and therefore the bioavailability of MPs for macroinvertebrates (Windsor et al., 2019). Accordingly, macroinvertebrates closest to the city have been shown to be more contaminated by MPs (Simmerman & Coleman Wasik, 2020). On the contrary, land use does not appear to influence MP concentration in the macroinvertebrates studied (Windsor et al., 2019). Most MP variability detected in macroinvertebrates is explained by each taxonomic group and biomass, although further investigation of biological traits is suggested (Windsor et al., 2019).

In addition to being ingested, MPs are used as a material to build casing (protective involucres) by caddisflies, suggesting a sequestering property (Ehlers et al., 2019; Tibbetts et al., 2018). This observation allows for speculation that MPs in the casing may impact larvae survival rates, for example by improving its visibility to predators or their drift. Further research is suggested to evaluate the interactions between wild biota and surrounding materials and their effects.

### 3.5 Vertebrates

#### 3.5.1 Fish

**FIGURE 3:**
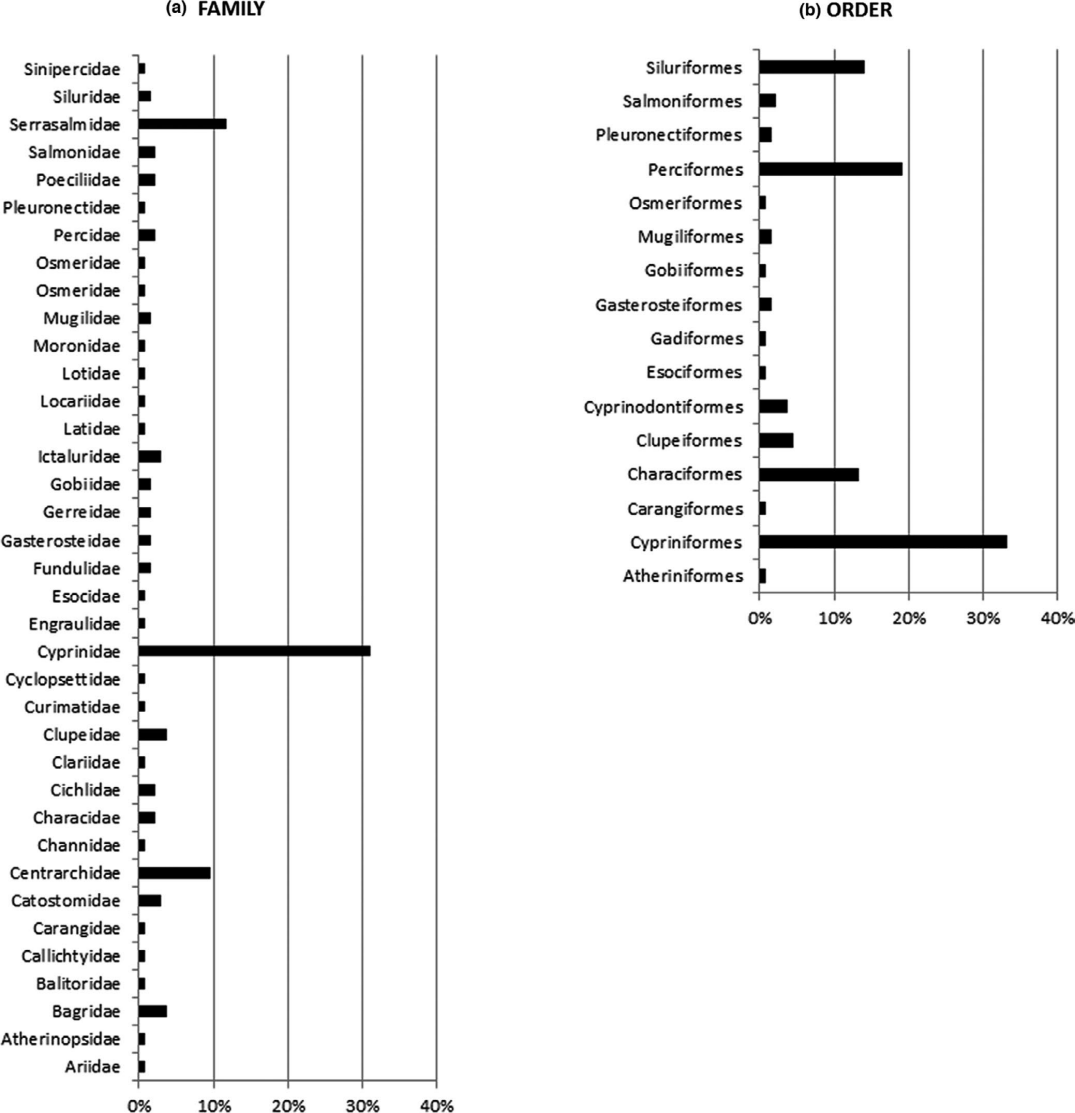
Frequency of fish Families (a) and Orders (b) examined by actual scientific literature on microplastics in fresh waters

Fish are at the center of research. 135 freshwater fish species are analyzed worldwide for MP contamination (Appendix S4). They belong to 37 families and 16 orders (Figure 3). *Cyprinus carpio* (Linnaeus, 1758) is the most investigated species, as it occurs in seven scientific articles (Appendix S4). The Family of Cyprinidae is the most investigated, including 31% of species (Figure 3a). The Order of Cypriniformes is the most studied (33%) (Figure 3b).

Most scientists collect field information from observation of gastrointestinal contents (GI). This is encouraged as Jabeen et al. (2017) observed that MPs are accumulated to a greater extent either in the stomach or the intestine based on fish species and therefore suggests sampling the entire GI. In addition, fish livers and muscles are rarely observed (Appendix S4).

The oldest study on the presence of MPs in fish GI was conducted in lentic fresh waters, in particular in Lake Geneva (Europe) in 2012. No MPs were found in fish intestines (Faure et al., 2012). However, in 2015, the specimens of Lake Geneva were analyzed again, finding 7.5% of plastic occurrence in their guts (Faure et al., 2015). In the same year, the first occurrence of MP ingestion was reported in Lake Victoria (Africa’s Great Lakes; Biginagwa et al., 2016). Their results show 20% contamination of the collected specimen, which is more than double the amount observed in Lake Geneva. The presence of fish contamination by MPs was also detected in Asian inland waters: In Lake Poyang, the occurrence of MPs in the gastrointestinal tract peaked by 90% and in Lake Qinghai by 100% (Xiong et al., 2018; Yuan et al., 2019). The different concentrations could be due to a geographical distribution of MPs as the Asian region is considered highly contaminated (Cera et al., 2020).

Regarding rivers, the first study was conducted on French rivers in 2013, detecting 13% of contaminated fish (Sanchez et al., 2014). Subsequently, other studies have evaluated the presence of fish contamination in Europe: for example, 33% in the River Thames and 9% in the Flemish rivers (Horton et al., 2018; Slootmaekers et al., 2019). Some studies have been conducted outside Europe, including Asia, for example, Xiangxi River (China) showing 25.7% contamination (Zhang et al., 2017), America, for example, Wascana Creek (Canada) and Pajeú River (Brazil) with a contamination of 73.5% and 83%, respectively (Campbell et al., 2017; Silva‐Cavalcanti et al., 2017), and Africa, for example, Nile River with a contamination of 76% and 79% (Khan et al., 2020). Similarly to the lacustrine results, it is suggested that the geographical location of the fish sampling influences the occurrence of the ingestion of MPs. This is a reasonable result, as MP pollution is not equally distributed worldwide (Cera et al., 2020).

In addition to the bioavailability of MP, the available scientific literature highlights various factors that influence the ingestion of MP, such as the morphology and feeding behavior of fish (Table 1). For instance, fish length correlates positively with the abundance of MPs in GI in some species (Peters & Bratton, 2016; Silva‐Cavalcanti et al., 2017), but not in others (McNeish et al., 2018). The accumulation of MPs in the GI is also evaluated according to the morphological characteristics of the GI itself. An increase in gut weight is observed to cause an increased presence of MPs (Jabeen et al., 2017; McNeish et al., 2018; Silva‐Cavalcanti et al., 2017). In fact, the presence of MPs correlates positively with the ingestion of food items, suggesting that the activity of feeding increases the chances of accidental MP ingestion (Peters & Bratton, 2016).

**TABLE 1:** Factors proposed to increase (+) or decrease (−) the probability of observing microplastics in fish gastrointestinal contents (GI)

Factor type	+ factor	− factor
MP bioavailability	environmental contamination	
Morphology	GI complexity	
	fish length	
trophy	benthic feeder	pelagic feeder
	zoobenthivore	detritivore
	eats glass	eats plants or algae
	food items occurring in the GI	eats fish
	GI weight	
	dominant food items	

The feeding behavior of the species is also likely to affect MP ingestion (Jabeen et al., 2017). Benthic feeders probably ingest more MPs than pelagic feeders (McGoran et al., 2017); zoo benthivores seem to ingest more MPs than detritivores and, in some cases, omnivores (McNeish et al., 2018); piscivorous fish are less affected by the ingestion of MPs (Roch et al., 2019). Moreover, the presence of MPs is negatively correlated with the abundance of plants or algae in the guts of fish, while it is positively correlated with the presence of glass items or dominant food items (Silva‐Cavalcanti et al., 2017).

The feeding guild is suggested to cause a difference in the ingestion of MPs (Hurt et al., 2020). However, a study of the Amazonian fish food web revealed no differences between the guilds of carnivores, herbivores, and omnivores (Andrade et al., 2019). A comprehensive pattern is not yet available. The theory that the presence of MPs in the GI is caused mostly by accidental uptake is supported by Roch et al. (2019), since in their study biotic and abiotic factors have a limited influence on the outcome of the ingestion.

To summarize, it seems that environmental contamination plays a fundamental role for GI contamination by MPs. The connection with the benthic niche may be a key factor positively influencing fish contamination (Merga et al., 2020). This could be explained by the observation that sediments are generally more contaminated than water (Cera et al., 2020). Furthermore, if the GI contains food and has complex morphology, the chances of detecting MPs are increased, probably because MPs are more likely to be ingested with food and to stay longer in the GI. Regarding the fact that increased fish length may reveal more MPs in the GI, we cannot exclude a methodological issue in species comparison: In fact, it has recently been suggested that larger MPs are found in longer fish (Jâms et al., 2020). This means that the MPs in larger fish are potentially more easily detectable than in smaller fish. Moreover, dietary changes due to ontogenesis may explain differences in the uptake of MPs by different specimens within the same species.

In addition to studies on ingestion, MPs in the livers and muscles of fish were investigated, in particular *Squalius cephalus* Linnaeus, 1758 from the Seine and Marne Rivers. The specimens were sampled upstream and downstream from the city of Paris (France), which represents an urban site with a high population density. Four MP fragments ranging from 147 to 567 μm were found in 3 of 60 livers analyzed, thus representing 5% of occurrence. The stomach contents (*n* = 60) were also analyzed from the same study, showing the presence of nine MPs. Therefore, the number of MPs for stomachs is 15% while for livers it is 5%. Roughly, the probability of finding MPs in stomachs is three times higher than that of finding it in livers (Collard et al., 2018). Furthermore, histological observations revealed changes in livers in MP‐contaminated areas compared with those obtained from a control area (Li, Busquets, et al., 2020; Li, Su, et al., 2020). The presence of MPs impacts in wild biota liver supports the need for laboratory research aiming at determining the presence and mechanisms of MP translocation and impacts on biota. In fact, some laboratory experiments indicate that MP microbeads or spheres do not translocate (De Sales‐Ribeiro et al., 2020; Kim et al., 2020).

Regarding fish muscles, 0 MPs were detected in 22 muscles of fish whose stomachs and livers were contaminated (Collard et al., 2018). MPs were absent in muscles even in a large South Korean river while ingestion and gill contamination were confirmed (Park et al., 2020).

#### 3.5.2 Amphibians

Few studies examine the ingestion of MPs by amphibians (Appendix S5). One study sampled 31 GI contents of different species of amphibians, all anurans: *Anaxyrus americanus* (Holbrook, 1836), *Lithobates clamitans* (Latreille, 1801), *Lithobates palustris* (LeConte, 1825), *Lithobates pipiens* (Schreber, 1782), and *Lithobates septentrionalis* (Baird, 1854); no plastic was found in their GI (Schessl et al., 2019). However, MPs were found in the diet of *Triturus carnifex* Laurenti, 1768 (Iannella et al., 2020) and tadpoles (Hu et al., 2018; Karaoğlu & Gül, 2020). Further evaluation of the occurrence of MPs in anurans and other amphibians is suggested. Given that amphibians are a globally endangered taxonomic group, understanding the potential impacts of MPs is a relevant research topic for their conservation.

#### 3.5.3 Reptiles and mammals

To date, no reptiles have been studied for MPs in fresh waters. The only study to have been made is on mammals in lotic fresh waters, where MPs have been detected in fecal samples of *Lutra lutra* (Linnaeus, 1758) (Smiroldo et al., 2019). We suggest increasing research on both taxa to assess their MP exposure and associated risk to organisms and food webs.

#### 3.5.4 Birds

The first bird record was conducted in 2012 (Appendix S6). It analyses only one specimen of *Podiceps nigricollis* Brehm, 1831 in Lake Geneva and finds no MPs in the GI (Faure et al., 2012). The same authors repeated the sampling in Lake Geneva, collecting 9 specimens belonging to the following species: *Anas platyrhynchos* L., 1758, *Ardea cinerea* L., 1758, and *Cygnus* *olor* (Gmelin, 1789). Eight out of nine birds had MPs in their GI (Faure et al., 2015). A larger number of specimens of *Cinclus cinclus* L., 1758 was sampled, using regurgitates and fecal samples to detect MP occurrence, which was found to be 50% and 45%, respectively (D’Souza et al., 2020). A larger study examines the ingestion of MPs from 350 specimens belonging to 17 species, including a marine one. They showed 11.1% ingestion of anthropogenic debris. Extrapolating data only on plastic, the occurrence of MPs in freshwater species is 9.7% (Holland et al., 2016). In addition to studies on adult birds, chicks of *Phalacrocorax auritus* (Lesson, 1831) from the Laurentian Great Lakes were dissected. Over 86% of the chicks had anthropogenic debris in their GI, mainly MP fibers (Brookson et al., 2019). From these preliminary studies, adult birds have less ingestion of MPs than juvenile forms, that is, chicks. However, specimens belonging to different ages of different species were collected, so this result could be due to a specific difference rather than to the stage of development.

## 4 KNOWLEDGE GAPS

### 4.1 Methodology

Microplastic sampling and analysis of results are the subject of intense standardization efforts in marine waters, thanks to specific projects such as the BASEMAN—JPI OCEANS (Bessa et al., 2019), and reports such as the “Guidelines for the monitoring and assessment of plastic litter in oceans” (GESAMP, 2019). In fresh waters, there is a lack of proposals for standardized protocols. However, recent reviews provide information on methodological aspects for water, sediments, and biota (Lu et al., 2021; O’Connor et al., 2019; Yang et al., 2021).

Some key points are the standardization of the units of measurement and the use of analytical techniques. As described above, different units provide different results in the classification of MP concentrations between species (Akindele et al., 2019). Other authors have pointed to the same issue while studying fish (Jabeen et al., 2017). It is highly recommended that this be taken into consideration when developing a standardized protocol for freshwater MPs. As some units are more used in the field and others in laboratory experiments, uniformity of units is also required for (O’Connor et al., 2019). This study highlights the most common units used by taxa for invertebrates and vertebrates (Appendices [Link], [Link], [Link], [Link], [Link]). Items/organisms and items/g of wet weight (ww) are the most common units of measurement for mollusks; it is suggested that both units be shown by authors. Regarding arthropods, there is no clear prevalence of a unit of measurement (Appendix S3). It is recommended to use items/organisms and items/g ww to standardize the results to mollusks. The same units could be applied to vertebrate species in order to uniform results from different trophic levels. However, fish and bird results are generally expressed as frequency of occurrence (Appendices S4 and S6). As calculating frequency of occurrence is usually not complicated, we suggest that this information be additionally reported in order to provide an opportunity to conduct comparisons with most articles. Moreover, many results are not available by species, but are shown cumulatively, so information is limited.

Analytical techniques such as Raman and FTIR are time‐consuming and expensive. However, it is suggested that their use be implemented in an attempt to identify all suspected MPs. The advantage is evident as the recent increase in the use of analytical techniques for the identification of polymers could have contributed to an increased detection of MPs in recent years. In fact, the first studies were based on techniques under stereomicroscope, such as the “hot needle”, while recent ones are based on the use of spectrophotometric techniques, thus identifying smaller MP items, which are usually more abundant than larger ones (Roch et al., 2019). In addition, the “hot needle” technique is based on the propriety of plastics to melt when heated; however, this melting propriety characterizes only a certain type of plastics, called thermoplastics. In contrast, plastics called thermosets do not melt when heated, so the “hot needle” technique does not work on them.

In conclusion, the adoption of a standardized protocol for detecting MPs in freshwater biota is required. This would make it possible to compare the results both geographically and temporally to monitor the trend of contamination and the suitability of policy actions.

### 4.2 Impacts on organisms

Field observations generally do not provide adequate information on the impacts of MPs on organisms. For instance, researchers are unable to detect the amount of fish or macroinvertebrates that have died due to MP ingestion as it is difficult to sample them. There is no doubt that laboratory experiments under controlled conditions could better analyze the processes that impact organisms. However, performing before‐after‐control‐impact studies on environmental concentrations of MPs and analyzing diachronic trends in population structure could provide cause–effect relationship information based on field information. The scientific literature lacks this type of articles and focuses mainly on the reporting of MP concentrations ingested by different species of invertebrates and vertebrates. Some articles also investigate the egestion, proving that MPs can be partially transient in the GI of organisms (Windsor et al., 2019). Some rare exceptions include the investigation of fish muscles and livers, and particularly, innovative is the histological analysis of liver stress. It is suggested that these studies be increased. If the liver histological alteration study is replicated (Li, Busquets, et al., 2020; Li, Su, et al., 2020), the stress observed in fish livers can be better understood, particularly if it is caused by different environmental factors or MPs. Instead, articles on microorganisms provide a wide range of observations which demonstrate that MPs can significantly alter the structure and functionality of communities in nature. The assessment of effects and potential ecological consequences is a significant knowledge gap based on the scientific literature collected.

### 4.3 Bioindicators of microplastics

Both vertebrates and invertebrates are evaluated as bioindicators of MPs in fresh waters. Among invertebrates, *Corbicula fluminea* (Müller, 1774) is proposed as a bioindicator of the concentration of MPs in environmental matrices, in particular in sediments as these are more closely related to this benthic species. *C*. *fluminea* has two main qualities to be a good bioindicator: It has a wide distribution and can accumulate MPs, especially small ones and fibers, according to environmental bioavailability, by filtering (Su et al., 2018). In a first study, *C*. *fluminea* led to the accumulation of MPs at low environmental concentrations with a higher degree than that found when MPs are more bioavailable (Su et al., 2016). A subsequent investigation was conducted, highlighting a positive correlation and a dependence between MP concentrations in *C*. *fluminea* and water and sediments, thus highlighting this organism as a good model (Su et al., 2018).

Regarding other invertebrates, *Chironomus* spp. are proposed as bioindicators of MPs because they accumulate MPs based on environmental concentrations. In particular, MPs were detected in the sediments of Bloukran River (South Africa) in winter rather than in summer. Similarly, *Chironomus* spp. contains a significant higher concentration of MPs in winter than in summer, showing a positive and significant correlation with the values of MPs in sediments. Therefore, *Chironomus* spp. and, more generally, deposit feeders, such as amphipods (Iannilli et al., 2020), are suggested as good bioindicators of the presence of MPs and of their abundance in running fresh waters (Nel et al., 2018).

In addition, the presence of MPs in fish gut could be a biomarker of environmental contamination, as suggested by Silva‐Cavalcanti et al. (2017). MP ingestion is affected by local conditions: For instance, if specimens are sampled near urbanized areas, they have more MPs in their guts (Peters & Bratton, 2016; Silva‐Cavalcanti et al., 2017). Fish biomonitoring was also conducted by sampling alien species, in particular the head and body of the mosquito fish *Gambusia holbrooki* Girard, 1859 in Australia (Su et al., 2019).

Among the proposed bioindicators, the relationship between MPs in the environment and MPs in fish is not yet clear, and invertebrates are therefore more suitable. It is suggested that *C*. *fluminea* and *Chironomus* spp. be evaluated in a comparative study in order to highlight the strengths and weaknesses of one taxon compared with the other and to evaluate the precision of results.

## 5 CONCLUSION

Microplastics affect freshwater biota worldwide and are a growing issue for management policies and monitoring activities. We have provided the scientific community with the first review on wild biota exposure to MPs exclusively in fresh waters.

Current scientific literature has mainly focused on freshwater lotic biota, so it is suggested that research on the biota of lentic ecosystems be increased. In addition, we highlight that about half of the current scientific literature examines the ingestion of MPs by fish. The ingestion of MPs by fish is a relevant research topic, given the information it could provide on MP exposure and the potential human impacts of consuming fish. However, our results and discussions indicate several interesting observations in other taxa, for instance the high ingestion of MPs by chicks, the use of MPs as construction material by caddisflies, and the functional diversity and richness of the freshwater plastisphere. Therefore, a disparity is reported between studies on fish and other taxa, which would require more investigation efforts for an assessment of MP exposure across the entire food web to provide information on the safety of the entire ecosystem. Moreover, there is a knowledge gap for plants, amphibian, mammals, and reptiles.

For monitoring activities, no comparison is made between deposit feeders, bivalves, and fish with regard to the efficiency of the bioindication of MPs. They could prove to be valuable monitoring tools and could be integrated based on species distribution ranges and local presence in the field. However, further investigations are mandatory. In this regard, evaluating and applying the few model organisms already proposed by the authors as monitoring tools before investigating new ones could help to avoid the redundant creation of numerous monitoring protocols leading to a lack of standardization, which is common in the scientific literature on MPs in fresh waters.

In conclusion, observation of wild biota provided valuable insight into freshwater MP exposure, demonstrating that a wide range of animals can be affected by MPs. It is suggested that further research be conducted to study the impacts on populations and communities. Moreover, the development of accurate biomonitoring tools could lower the costs of sampling and analyzing water and sediment. However, in contributing to solving the issue of MPs, research, policies, and funding that enable proper wastewater treatment are likely to reduce biota contamination by MPs by improving overall water quality and ecosystem health.

## CONFLICT OF INTEREST

The authors declare no conflicts of interest.

## AUTHOR CONTRIBUTIONS

**Alessandra Cera:** Conceptualization (lead); Data curation (lead); Formal analysis (lead); Investigation (lead); Methodology (lead); Resources (equal); Software (lead); Validation (equal); Visualization (equal); Writing‐original draft (lead); Writing‐review & editing (equal). **Massimiliano Scalici:** Funding acquisition (lead); Project administration (lead); Resources (equal); Supervision (lead); Validation (equal); Visualization (equal); Writing‐review & editing (equal).

## Supporting information

Appendix S1

Appendix S2

Appendix S3

Appendix S4

Appendix S5

Appendix S6

## Data Availability

Data are available from Appendix S1.
