# Supplementary material for: Freshwater wild biota exposure to microplastics: A global perspective
Source: Ecol Evol. 2021 Jul 9;11(15):9904–16. doi: 10.1002/ece3.7844 (PMC8328441; doi:10.1002/ece3.7844)
Supplement: Supplementary file 1 — Appendix S1 [file ECE3-11-9904-s001.docx]

**Appendix 1.** List of scientific articles on wild biota in freshwater. The type of investigated freshwater (lentic/lotic) and taxa are reported.

| **Article reference** | **Microplastics investigated in:** | | | | | | | | | |
| --- | --- | --- | --- | --- | --- | --- | --- | --- | --- | --- |
|  | **Prokaryota - Protista and Chromista** | **Plantae (Chlorophyta)** | **Fungi** | **Invertebrates** | | **Fish** | | **Amphibia** | **Birds** | **Mammals** |
| **Lentic freshwater** |  |  |  | |  | |  |  |  |  |
| Arias-Andres et al., 2018 | x |  |  | |  | |  |  |  |  |
| Baldwin et al., 2020 |  |  |  | | x | | x |  |  |  |
| Biginagwa et al., 2015 |  |  |  | |  | | x |  |  |  |
| Brookson et al., 2019 |  |  |  | |  | |  |  | x |  |
| Chen et al., 2019 | x | x |  | |  | |  |  |  |  |
| Faure et al., 2012 |  |  |  | |  | | x |  | x |  |
| Faure et al., 2015 |  |  |  | |  | | x |  | x |  |
| Hu et al., 2018 |  |  |  | |  | |  | x |  |  |
| Hurt et al., 2020 |  |  |  | |  | | x |  |  |  |
| Iannella et al., 2020 |  |  |  | |  | |  | x |  |  |
| Iannilli et al., 2020 |  |  |  | | x | |  |  |  |  |
| Jabeen et al., 2017 |  |  |  | |  | | x |  |  |  |
| Karaoğlu and Gül, 2020 | |  |  | |  | |  | x |  |  |
| Leiser et al., 2020 | x |  |  | |  | |  |  |  |  |
| Merga et al., 2020 |  |  |  | |  | | x |  |  |  |
| Roch et al., 2019* |  |  |  | |  | | x |  |  |  |
| Su et al., 2016 |  |  |  | | x | |  |  |  |  |
| Su et al., 2018* |  |  |  | | x | |  |  |  |  |
| Su et al., 2019 |  |  |  | |  | | x |  |  |  |
| Xiong et al., 2018 |  |  |  | |  | | x |  |  |  |
| Xu et al., 2020 |  |  |  | | x | |  |  |  |  |
| Yuan et al., 2019 |  |  |  | |  | | x |  |  |  |
|  |  |  |  | |  | |  |  |  |  |
| **Lotic freshwater** |  |  |  | |  | |  |  |  |  |
| Akindele et al., 2019 |  |  |  | | x | |  |  |  |  |
| Andrade et al., 2019 |  |  |  | |  | | x |  |  |  |
| Berglund et al., 2019 |  |  |  | | x | |  |  |  |  |
| Campbell et al., 2017 |  |  |  | |  | | x |  |  |  |
| Collard et al., 2018 |  |  |  | |  | | x |  |  |  |
| D'Souza et al., 2020 |  |  |  | |  | |  |  | x |  |
| Domogalla-Urbansky et al., 2019 | |  |  | | x | |  |  |  |  |
| Ehlers et al., 2019 |  |  |  | | x | |  |  |  |  |
| Hoellein et al., 2017 | x |  |  | |  | |  |  |  |  |
| Horton et al., 2018 |  |  |  | |  | | x |  |  |  |
| Hurley et al., 2017 |  |  |  | | x | |  |  |  |  |
| Kettner, 2017 |  |  | x | |  | |  |  |  |  |
| Kettner, 2019 | x |  |  | |  | |  |  |  |  |
| Khan et al., 2020 |  |  |  | |  | | x |  |  |  |
| Li et al., 2020 |  |  |  | |  | | x |  |  |  |
| McCormick et al., 2014 | x |  |  | |  | |  |  |  |  |
| McCormick et al., 2016 | x |  |  | |  | |  |  |  |  |
| McCoy et al., 2020 |  |  |  | | x | |  |  |  |  |
| McGoran et al., 2017 |  |  |  | |  | | x |  |  |  |
| McNeish et al., 2018 |  |  |  | |  | | x |  |  |  |
| Nan et al., 2020 |  |  |  | | x | |  |  |  |  |
| Nel et al., 2018 |  |  |  | | x | |  |  |  |  |
| Oberbeckmann et al., 2018 | x |  |  | |  | |  |  |  |  |
| Park et al., 2020 |  |  |  | |  | | x |  |  |  |
| Pazos et al., 2017 |  |  |  | |  | | x |  |  |  |
| Peters and Bratton, 2016 | |  |  | |  | | x |  |  |  |
| Phillips and Bonner, 2015 | |  |  | |  | | x |  |  |  |
| Roch et al., 2019* |  |  |  | |  | | x |  |  |  |
| Ryan et al., 2019 |  |  |  | |  | | x |  |  |  |
| Sanchez et al., 2014 |  |  |  | |  | | x |  |  |  |
| Schessl et al., 2019 |  |  |  | | x | |  | x |  |  |
| Silva-Cavalcanti et al., 2017 | |  |  | |  | | x |  |  |  |
| Simmerman and Coleman Wasik, 2020 | | |  | | x | | x |  |  |  |
| Slootmaekers et al., 2019 | |  |  | |  | | x |  |  |  |
| Smiroldo et al., 2019 | |  |  | |  | |  |  |  | x |
| Su et al., 2018* |  |  |  | | x | |  |  |  |  |
| Thaysen et al., 2020 |  |  |  | |  | |  |  | x |  |
| Tibbetts et al., 2018 |  |  |  | | x | |  |  |  |  |
| Vendel et al., 2017 |  |  |  | |  | | x |  |  |  |
| Windsor et al., 2019 |  |  |  | | x | |  |  |  |  |
| Zhang et al., 2017 |  |  |  | |  | | x |  |  |  |
| Holland et al., 2016** |  |  |  | |  | |  |  | x |  |

* = same article; **=not specified if lentic or lotic
