# Supplementary material for: Freshwater wild biota exposure to microplastics: A global perspective
Source: Ecol Evol. 2021 Jul 9;11(15):9904–16. doi: 10.1002/ece3.7844 (PMC8328441; doi:10.1002/ece3.7844)
Supplement: Supplementary file 2 — Appendix S2 [file ECE3-11-9904-s004.docx]

**Appendix 2.** Focus of the investigations on microplastics in species of molluscs.

| **Species** | **Analised component** | **Results** | **Particle size** | **Polymer types** | **Morphology** | **Analytical method** | **Reference** |
| --- | --- | --- | --- | --- | --- | --- | --- |
| *Anodonta anatina* Linnaeus, 1758 | soft tissue | 75% occurrence, 2-71 items/organism |  |  | fibres (100%) | hot needle | Berglund et al., 2019 |
| *Bellamya aeruginosa* (Reeve, 1863) | soft tissue | 96.7-100% of occurrence, 6.1 ± 2.0 items/organism, 5.8 ± 2.0 items/g wet weight | 50-810 μm, 264 ± 154 μm | poly(vinyl acetate) (88.0 ± 12.1%), PS (66.3 ± 17.5%), PA (49.7 ± 22.4%), PET (30.0 ± 7.4%) | fibre most frequent than fragments | micro-FTIR | Xu et al., 2020 |
| *Corbicula fluminea* (Müller, 1774) | soft tissue | 6.40 items/organism | < 5 mm | several, mainly PP (57%) | all fibres apart from 2 films | FTIR (subsample) | McCoy et al. 2020 |
|  | soft tissue | 0.2–12.5 items/g ww, August: 1.3–12.5 items/g ww, November: 0.2–9.6 items/g ww | main class size: 333-1000 μm | not available by species | fibres>fragment>pellet>film | micro-FTIR or SEM-EDS | Su et al., 2016 |
|  | soft tissue | 0.3-4.9 items/g, 0.4-5.0 items/organism | 0.021-4.02 mm, mostly 0.25-1 mm | not available by species | fibres (60-100%) | micro-FTIR | Su et al., 2018 |
| *Dreissena bugensis* (*Dreissena rostriformis bugensis* Andrusov, 1897) | soft tissue | Site 1: 13.0 items/organism, site 2: 2.7 items/organism | < 5 mm |  | not available by species | steromicroscope | Baldwin et al. 2020 |
|  | soft tissue | 0 microbeads |  |  |  | steromicroscope | Schessl et al., 2019 |
| *Dreissena polymorpha* (Pallas, 1766) | soft tissue | 0 microbeads |  |  |  | steromicroscope | Schessl et al., 2019 |
| *Lanistes varicus* (Müller, 1774) | soft tissue | 1.71 ± 0.46 items/g ww, 3.8 items/organisms (data extrapolated from histogram) |  |  | fibres (65.8%) | ATR-micro-FTIR | Akindele et al., 2019 |
| *Melanoides tuberculata* (Müller, 1774) | soft tissue | 4.5 items/g ww (data extrapolated from histogram), 1.8 items/organisms |  |  | fibres (100%) | ATR-micro-FTIR | Akindele et al., 2019 |
| *Theodoxus fluviatilis* (Linnaeus, 1758) | soft tissue | 6.1 ± 1.05 items/g ww, 0.5 items/organisms (data extrapolated from histogram) |  |  | fibres (100%) | ATR-micro-FTIR | Akindele et al., 2019 |
| *Unio pictorum* Linnaeus, 1758 | soft tissue | 28 days exposure: 2 microplastics, 6 months exposure: 9 microplastics | 28 days exposure: > 50 μm, 6 months exposure: 1 microplastic > 50 μm & 8 microplastics < 50 μm | 28 days exposure: 1 PP, 1 PET, 6 months exposure: 2 PP, 7 PVC | particles, fibres | Raman (subsample) | Domogalla-Urbansky et al., 2019 |
