# Supplementary material for: Freshwater wild biota exposure to microplastics: A global perspective
Source: Ecol Evol. 2021 Jul 9;11(15):9904–16. doi: 10.1002/ece3.7844 (PMC8328441; doi:10.1002/ece3.7844)
Supplement: Supplementary file 3 — Appendix S3 [file ECE3-11-9904-s006.docx]

**Appendix 3.** Focus of the investigations on microplastics in species of anthropods.

| **Taxa** | **Analised component** | **Results** | **Particle size** | **Polymer types** | **Morphology** | **Analytical method** | **Reference** |
| --- | --- | --- | --- | --- | --- | --- | --- |
| Baetidae | pool of organisms | 0.02 items/mg (data extrapolated from histogram) |  |  |  | microscope | Windsor et al., 2019 |
| Chironomidae | pool of organisms | summer: 75% of occurrence, 0.37 ± 0.44 items/mg ww, winter: 98% of occurrence, 1.12 ± 1.19 items/mg ww |  |  |  | stereomicroscope | Nel et al., 2018 |
| Gammaridae | pool of organisms | 100% of occurrence | not available by taxa |  | fragments and fibres | hot needle | Simmerman and Coleman Wasik, 2020 |
| *Gammarus setosus* Dementieva, 1931 | digestive tract | average 72.5 items/organism, range 65-90 items/organism | 3-370 μm, 76% < 30 μm, average 25.73 μm | poly-methyl-acrylamide (81.17%), polyacrylamide, nylon | fragments (95%), fibres (5%) | Nile red staining or micro-FTIR (subsample) | Iannilli et al., 2020 |
| Heptageniidae | pool of organisms | 0.02 items/mg (data extrapolated from histogram) |  |  |  | microscope | Windsor et al., 2019 |
|  | pool of organisms | 100% of occurrence | not available by taxa |  | fragments and fibres | hot needle | Simmerman and Coleman Wasik, 2020 |
| Hydropsychidae | pool of organisms | 0.02 items/mg (data extrapolated from histogram) |  |  |  | microscope | Windsor et al., 2019 |
|  | pool of organisms | 100% of occurrence | not available by taxa |  | fragments and fibres | hot needle | Simmerman and Coleman Wasik, 2020 |
| *Lepidostoma basale* (Kolenati, 1848) | case | 59% of occurrence, 1.14 ± 0.28 items/case, range 0-6 items/case, 0.36 ± 0.09 items/mg | fragments: 236.09 ± 33.44 µm, films: 98.92 ± 18.57 µm, spheres: 91.20 ± 25.88 µm, fibres: 1872.40 ± 1434.03 µm | PP, PA, ABS, TPU, vynil ester resin, PVC, PE, PS |  | ATR-micro-FTIR | Ehlers et al., 2019 |
| *Paratya australiensis* Kemp, 1917 | whole individual | 36% of occurrence, 0.52 ± 0.55 items/organism, 24 ± 31 items/g | 0.190-4.214 mm | rayon (22.6), PE (7.5%), 9 other types rarer | all fibres except 1 fragment | ATR-micro-FTIR | Nan et al., 2020 |
| Trichoptera | case | 1 microplastic | about 1 mm |  | fragment | streomicroscope | Tibbetts et al., 2018 |
