# Supplementary material for: Freshwater wild biota exposure to microplastics: A global perspective
Source: Ecol Evol. 2021 Jul 9;11(15):9904–16. doi: 10.1002/ece3.7844 (PMC8328441; doi:10.1002/ece3.7844)
Supplement: Supplementary file 4 — Appendix S4 [file ECE3-11-9904-s003.docx]

**Appendix 4.** Focus of the investigations on microplastics in freshwater fish species.

| **Species** | **Analised component** | **Results** | **Particle sizes** | **Polymer types** | **Morphology** | **Analytical method** | **Reference** |
| --- | --- | --- | --- | --- | --- | --- | --- |
| *Abramis brama* Linnaeus, 1758 | Gut contents | 0 microplastics |  |  |  |  | Faure et al. 2012 |
|  | Gastrointestinal contents | 0 microplastics |  |  |  |  | Roch et al. 2019 |
| *Acnodon normani* Gosline 1951 | Stomach contents | 0 microplastics |  |  |  |  | Andrade et al. 2019 |
| *Alburnus alburnus* Linnaeus, 1758 | Gut contents | 31 microplastics  total weight 0.2 mg | < 5 mm |  | fibers (100%) | steromicroscope | Faure et al. 2015 |
|  | Gastrointestinal contents | 17% of occurrence (data extrapolated from histogram) | not available by species |  | not available by species | hot needle | Roch et al. 2019 |
| *Ameiurus melas* (Rafinesque 1820) | Gut contents | 0 microplastics |  |  |  |  | Phillips and Bonner, 2015 |
| *Ameiurus natalis* (Lesueur 1819) | Gut contents | microplastics present | not available by species |  | not available by species | FTIR (subsample) | Phillips and Bonner, 2015 |
| *Astyanax mexicanus* (De Filippi 1853) | Gut contents | microplastics present | not available by species |  | not available by species | FTIR (subsample) | Phillips and Bonner, 2015 |
| *Astyanax rutilus* (Jenyns, 1842) | Gut contents | not available by species | < 5 mm | not available by species | not available by species | stereomicroscope | Pazos et al. 2017 |
| *Bagrus bajad* Forskål, 1775 | Gastrointestinal contents | 78.6% of occurrence  4.7 ± 1.7 items/fish | < 5 mm | not available by species | fibers (61.7%), films (29.8%), fragments (8.5%) | ATR-FTIR (subsample) | Khan et al. 2020 |
| *Barbatula barbatula* (Linnaeus, 1758) | Gastrointestinal contents | 17% of occurrence (data extrapolated from histogram) | not available by species |  | not available by species | hot needle | Roch et al. 2019 |
| *Blicca bjoerkna* (Linnaeus, 1758) | Gastrointestinal contents | 25% of occurrence (data extrapolated from histogram) | not available by species |  | not available by species | hot needle | Roch et al. 2019 |
| *Brevoortia patronus* Goode, 1878 | Gut contents | 0 microplastics |  |  |  |  | Phillips and Bonner, 2015 |
| *Campostoma anomalum* (Rafinesque 1820) | Gut contents | microplastics present | not available by species |  | not available by species | FTIR (subsample) | Phillips and Bonner, 2015 |
| *Caranx hippos* (Linnaeus 1766) | Gut contents | 0 microplastics |  |  |  |  | Phillips and Bonner, 2015 |
| *Carassius auratus* (Linnaeus, 1758) | Gastrointestinal contents | 1.7±1.0 items/g  1.9±1.0 items/fish | < 5 mm | not available by species | fibers (86.5%)  pellet (5.4%) | micro-FTIR | Jabeen et al. 2017 |
|  | Gastrointestinal contents | 90% of occurrence  range 0-18 items/fish | 82.1% larger than 0.5 mm  14.3% larger than 5 mm | not available | fibers are the most common | micro-Raman | Yuan et al. 2019 |
|  | Gastrointestinal contents | 0 microplastics |  |  |  |  | Li et al. 2020 |
| *Carassius carassius* (Linnaeus, 1758) | Gastrointestinal contents | 61% of occurrence | < 5 mm | not available by species | not available by species | ATR- FTIR (subsample) | Merga et al. 2020 |
| *Carassius cuvieri* Temminck & Schlegel, 1846 | Gastrointestinal contents, gills | gastointestine: 4 microplastics  gills: 1 microplastic | < 5 mm | not available by species | not available by species | FTIR | Park et al. 2020 |
| *Carpoides cyprinus* (Lesueur, 1817) | Gastrointestinal contents | 14 items/fish (data extrapolated from histogram) | < 5 mm |  |  | FTIR (subsample) | McNeish et al. 2018 |
| *Catostomus commersonii* (Lacépède, 1803) | Gastrointestinal contents | 70% of occurrence (data extrapolated from histogram) | < 5 mm |  |  | stereomicroscope | Campbell et al. 2017 |
|  | Gastrointestinal contents | 7 items/fish (data extrapolated from histogram) | < 5 mm |  |  | FTIR (subsample) | McNeish et al. 2018 |
| *Channa argus* (Cantor, 1842) | Gastrointestinal contents, gills | gastointestine: 32 microplastics  gills: 6 microplastics | < 5 mm |  | not available by species | FTIR | Park et al. 2020 |
| *Citharichthys spilopterus* Günther 1862 | Gastrointestinal contents | 0 items/fish | < 5 mm |  | not available by species | stereomicroscope | Vendel et al. 2017 |
| *Clarias gariepinus* (Burchell 1822) | Gastrointestinal contents | 71% of occurrence | < 5 mm | not available by species | not available by species | ATR- FTIR (subsample) | Merga et al. 2020 |
| *Coregonus wartmanni* (Bloch, 1784) | Gastrointestinal contents | 35% of occurrence (data extrapolated from histogram) | not available by species |  | not available by species | hot needle | Roch et al. 2019 |
| *Ctenogobius boleosoma* (Jordan & Gilbert 1882) | Gut contents | 0-0.06 items/fish | < 5 mm |  | not available by species | stereomicroscope | Vendel et al. 2017 |
| *Culter alburnus* Basilewsky, 1855 | Gastrointestinal contents | 1.5±1.38 items/fish | 0.3-0.5 mm | PE | lines | Raman | Zhang et al. 2017 |
| *Culter dabryi* Bleeker, 1871 | Gastrointestinal contents | 0.5±0.71 items/fish | 1 mm | PE | film | Raman | Zhang et al. 2017 |
| *Culter mongolicus* (Basilewsky, 1855) | Gastrointestinal contents | 0 microplastics |  |  |  |  | Zhang et al. 2017 |
| *Culter oxycephaloides* Kreyenberg & Pappenheim, 1908 | Gastrointestinal contents | 0 microplastics |  |  |  |  | Zhang et al. 2017 |
| *Cyphocharax voga* (Hensel, 1836) | Gut contents | not available by species | < 5 mm | not available by species | not available by species | stereomicroscope | Pazos et al. 2017 |
| *Cyprinella lepida* Girard 1856 | Gut contents | 0 microplastics |  |  |  |  | Phillips and Bonner, 2015 |
| *Cyprinella lutrensis* (Baird & Girard 1853) | Gut contents | microplastics present | not available by species |  | not available by species | FTIR (subsample) | Phillips and Bonner, 2015 |
| *Cyprinella spiloptera* (Cope, 1867) | Gastrointestinal contents | 10 items/fish (data extrapolated from histogram) |  |  |  | FTIR (subsample) | McNeish et al. 2018 |
| *Cyprinella venusta* Girard 1856 | Gut contents | microplastics present | not available by species |  | not available by species | FTIR (subsample) | Phillips and Bonner, 2015 |
| *Cyprinus carpio* (Linnaeus, 1758) | Gastrointestinal contents | 0.5±0.3 items/g  2.5±1.3 items/fish | < 5 mm | not available by species | fibers (83.3%) | micro-FTIR | Jabeen et al. 2017 |
|  | Gastrointestinal contents | 0 microplastics |  |  |  |  | Zhang et al. 2017 |
|  | Gastrointestinal contents, gills, muscle | gastointestine: 48 microplastics  gills: 8 microplastics  muscle: 0 microplastics | < 5 mm | not available by species | not available by species | FTIR | Park et al. 2020 |
|  | Gastrointestinal contents | 0 microplastics |  |  |  |  | Li et al. 2020 |
|  | Gut contents | not available by species | < 5 mm | not available by species | not available by species | stereomicroscope | Pazos et al. 2017 |
|  | Gastrointestinal contents | 11 items/fish (mean)  12 items/fish (median) | < 5 mm |  | not available by species | stereomicroscope | Baldwin et al. 2020 |
|  | Gastrointestinal contents | 69% of occurrence | < 5 mm | not available by species | not available by species | ATR- FTIR (subsample) | Merga et al. 2020 |
| *Dorosoma cepedianum* (Lesueur, 1818) | Gastrointestinal contents | 0 microplastics |  |  |  |  | McNeish et al. 2018 |
|  | Gut contents | microplastics present | not available by species |  | not available by species | FTIR (subsample) | Phillips and Bonner, 2015 |
|  | Gut contents, gills | 100% of occurrence  gut: 2 items/fish  gills: 3 items/fish (data extrapolated from histogram) | < 5 mm |  |  | hot needle (subsample) | Hurt et al. 2020 |
| *Dorosoma petenense* (Günther 1867) | Gut contents | microplastics present | not available by species |  | not available by species | FTIR (subsample) | Phillips and Bonner, 2015 |
| *Erimyzon oblongus* (Mitchill 1814) | Gut contents | 0 microplastics |  |  |  |  | Phillips and Bonner, 2015 |
| *Esox lucius* Linnaeus, 1758 | Gut contents | 0 microplastics |  |  |  |  | Faure et al. 2012 |
|  | Gastrointestinal contents | 83.3% of occurrence |  |  |  | stereomicroscope | Campbell et al. 2017 |
|  | Gastrointestinal contents | 10% of occurrence (data extrapolated from histogram) | not available by species |  | not available by species | hot needle | Roch et al. 2019 |
| *Etheostoma artesiae* (Hay 1881) | Gut contents | microplastics present | not available by species |  | not available by species | FTIR (subsample) | Phillips and Bonner, 2015 |
| *Eucalia inconstans* Jordan, 1876 | Gastrointestinal contents | 70% of occurrence (data exptrapoleted from histogram) |  |  |  |  | Campbell et al. 2017 |
| *Eucinostomus argenteus* Baird & Girard 1855 | Gastrointestinal contents | 0-0.2 items/fish | < 5 mm |  | not available by species | stereomicroscope | Vendel et al. 2017 |
| *Eucinostomus melanopterus* (Bleeker 1863) | Gastrointestinal contents | 0 items/fish |  |  |  |  | Vendel et al. 2017 |
| *Fundulus diaphanus* (Lesueur, 1817) | Gastrointestinal contents | 10 items/fish | < 5 mm |  |  | FTIR (subsample) | McNeish et al. 2018 |
| *Fundulus notatus* (Rafinesque 1820) | Gut contents | microplastics present | not available by species |  | not available by species | FTIR (subsample) | Phillips and Bonner, 2015 |
| *Gambusia affinis* (Baird & Girard 1853) | Gut contents | microplastics present | not available by species |  | not available by species | FTIR (subsample) | Phillips and Bonner, 2015 |
| *Gambusia holbrooki* Girard, 1859 | Head, body | head: 7.2% of occurrence  0.1 items/fish  3.1 items/g  body: 19.4% of occurrence  0.6 items/fish  1.94 items/g | head: 0.22-2.01 mm  < 1 mm 83.9%  body: 0.09-4.86 mm  < 1 mm 52.9% | PE (25.7%), rayon (10.1%), PA (7.3%), PP (5.5%) | fibers (62.5%-100%) | ATR-micro-FTIR (subsample) | Su et al. 2019 |
| *Gastosterus aculeatus* Linnaeus, 1758 | Gastrointestinal contents | 20% of occurrence (data extrapolated from histogram) | not available by species |  | not available by species | hot needle | Roch et al. 2019 |
| *Gobio gobio* (Linnaeus, 1758) | Gastrointestinal contents | 12% of occurrence | < 5 mm |  |  | stereomicroscope | Sanchez et al. 2014 |
|  | Gastrointestinal contents | 8 microplastics  9% of occurrence | 0.08-3.4 mm | PET (detected twice), PP, PVC, PA, ethylene-vynil acetate copolymer, cellophane, polyvinyl acetate | foam (25%), film (25%), fibre (25%), fragment (12.5%), pellet (12.5%) | micro-FTIR | Slootmaekers et al. 2019 |
| *Gymnocephalus cernua* (Linnaeus, 1758) | Gastrointestinal contents | 17% of occurrence (data extrapolated from histogram) | not available by species |  | not available by species | hot needle | Roch et al. 2019 |
| *Gymnocypris przewalskii* (Kessler, 1876) | Gastrointestinal contents | 5.4±3.6 (2-15) items/fish | < 5 mm | PE, PS, nylon, PP | occurrence of fibres (100%) and sheets (50%) | Raman (subsample) | Xiong et al. 2018 |
| *Hemiculter bleekeri* (Warpachowski, 1888) | Gastrointestinal contents | 1.1±0.5 items/g  2.1±1.1 items/fish | < 5 mm | not available by species | fibers (88.2%) | micro-FTIR | Jabeen et al. 2017 |
| *Hemiculter leucisculus* (Basilewsky, 1855) | Gastrointestinal tract, liver | - microplastics   site “P”: 1.9-6.1 items/fish  2.3-15.8 items/g of digestive tissue  site “R”: 0.2±0.01 items/fish  0.3±0.1 items/g of digestive tissue   - organ index of fish liver   site “P”: 5.53-9.91  site “R”: 1.63± | 49% < 1 mm  95% < 3 mm | not available | fiber (86%)  fregment (14%) | micro-FTIR (subsample) | Li et al. 2020 |
| *Herichthys cyanoguttatus* Baird & Girard 1854 | Gut contents | microplastics present | not available by species |  | not available by species | FTIR (subsample) | Phillips and Bonner, 2015 |
| *Hoplosternum littorale* (Hancock, 1828) | Gut contents | 3.25 items/fish | < 5 mm |  | fibers (46.6%)  “soft” plastic (36%)  “hard” plastic (17.4%) | stereomicroscope | Silva-Cavalcanti et al. 2017 |
| *Hypophthalmichthys molitrix* (Valenciennes, 1844) | Gastrointestinal contents | 2.1±1.1 items/g  3.8±2.0 items/fish | < 5 mm | not available by species | fibers (57.6%)  fragments (6.5%)  pellets (9.8%) | micro-FTIR | Jabeen et al. 2017 |
|  | Gastrointestinal contents | 0 microplastics |  |  |  |  | Li et al. 2020 |
| *Hypostomus commersoni* Valenciennes, 1836 | Gut contents | not available by species | < 5 mm | not available by species | not available by species | stereomicroscope | Pazos et al. 2017 |
| *Ictalurus punctatus* (Rafinesque 1818) | Gut contents | microplastics present | not available by species |  | not available by species | FTIR (subsample) | Phillips and Bonner, 2015 |
| *Lates niloticus* (Linnaeus, 1758) | Gastrointestinal contents | 20% of occurrence | < 5 mm | not available by species |  | ATR-FTIR (subsample) | Biginagwa et al. 2015 |
| *Lepomis auritus* (Linnaeus 1758) | Gut contents | microplastics present | not available by species |  | not available by species | FTIR (subsample) | Phillips and Bonner, 2015 |
| *Lepomis cyanellus* Rafinesque 1819 | Gut contents | microplastics present | not available by species |  | not available by species | FTIR (subsample) | Phillips and Bonner, 2015 |
| *Lepomis gulosus* (Cuvier 1829) | Gut contents | 0 microplastics |  |  |  |  | Phillips and Bonner, 2015 |
| *Lepomis humilis* (Girard 1858) | Gut contents | microplastics present | not available by species |  | not available by species | FTIR (subsample) | Phillips and Bonner, 2015 |
| *Lepomis macrochirus* Rafinesque, 1810 | Stomach contents | 45% of occurrence | < 5 mm |  |  | stereomicroscope | Peters and Bratton, 2016 |
|  | Gastrointestinal contents, gills | gastointestine: 10 microplastics  gills: 16 microplastic | < 5 mm | not available by species | not available by species | FTIR | Park et al. 2020 |
|  | Gut contents | microplastics present | not available by species |  | not available by species | FTIR (subsample) | Phillips and Bonner, 2015 |
| *Lepomis megalotis* (Rafinesque, 1820) | Stomach contents | 44% of occurrence | < 5 mm |  |  | stereomicroscope | Peters and Bratton, 2016 |
| *Lepomis microlophus* (Günther 1859) | Gut contents | microplastics present | not available by species |  | not available by species | FTIR (subsample) | Phillips and Bonner, 2015 |
| *Lepomis miniatus* (Jordan 1877) | Gut contents | 0 microplastics |  |  |  |  | Phillips and Bonner, 2015 |
| *Leuciscus leuciscus* (Linnaeus, 1758) | Gut contents | 3 microplastics  total weight 0.4 mg | < 5 mm |  | fragments (100%) | steromicroscope | Faure et al. 2015 |
|  |  | 0 microplastics |  |  |  |  | Roch et al. 2019 |
| *Lota lota* (Linnaeus, 1758) | Gastrointestinal contents | 20% of occurrence (data extrapolated from histogram) | not available by species |  | not available by species | hot needle | Roch et al. 2019 |
| *Luciopimelodus pati* (Valenciennes, 1836) | Gut contents | not available by species | < 5 mm | not available by species | not available by species | stereomicroscope | Pazos et al. 2017 |
| *Lycengraulis grossidens* (Spix & Agassiz 1829) | Gut contents | 0.17-0.20 items/fish | < 5 mm |  | not available by species | stereomicroscope | Vendel et al. 2017 |
| *Megalobrama amblycephala* (Yih, 1955) | Gastrointestinal contents | 0.2±0.1 items/g  1.8±1.7 items/fish | < 5 mm | not available by species | fibers (26.3%)  fragments (10.5%) | micro-FTIR | Jabeen et al. 2017 |
| *Metynnis guaporensis* Eigenmann, 1915 | Stomach contents | 0 microplastics |  |  |  |  | Andrade et al. 2019 |
| *Metynnis luna* Cope, 1878 | Stomach contents | 0 microplastics |  |  |  |  | Andrade et al. 2019 |
| *Micropterus* Lacepède, 1802 sp. | Gastrointestinal contents | 14 items/fish (data extrapolated from histogram) | < 5 mm |  |  | FTIR (subsample) | McNeish et al. 2018 |
| *Minytrema melanops* (Rafinesque 1820) | Gut contents | 0 microplastics |  |  |  |  | Phillips and Bonner, 2015 |
| *Micropterus punctulatus* (Rafinesque, 1819) | Gut contents | 0 microplastics |  |  |  |  | Phillips and Bonner, 2015 |
| *Micropterus salmoides* Lacepède, 1802 | Gut contents, gills | 100% of occurrence  gut: 15 items/fish  gills: 10 items/fish (data extrapolated from histogram) | < 5 mm |  |  | hot needle (subsample) | Hurt et al. 2020 |
|  | Gastrointestinal contents, gills | gastointestine: 16 microplastics  gills: 4 microplastic | < 5 mm | not available by species | not available by species | FTIR | Park et al. 2020 |
|  | Gut contents | microplastics present | not available by species |  | not available by species | FTIR (subsample) | Phillips and Bonner, 2015 |
| *Morone saxatilis* (Walbaum, 1792) | Gastrointestinal contents | 4.2 items/fish (mean)  2.0 items/fish (median) | < 5 mm |  | not available by species | stereomicroscope | Baldwin et al. 2020 |
| *Myloplus asterias* (Müller & Troschel, 1844) | Stomach contents | 0 microplastics |  |  |  |  | Andrade et al. 2019 |
| *Myloplus rhomboidalis* (Cuvier, 1818) | Stomach contents | 0 microplastics |  |  |  |  | Andrade et al. 2019 |
| *Myloplus rubripinnis* (Müller and Troschel, 1844) | Stomach contents | 2 microplastics | 2.0 - 4.9 mm  3.5±2 mm | not available by species | not available by species | ATR-FTIR (subsample) | Andrade et al. 2019 |
| *Myloplus schomburgkii* (Jardine, 1841) | Stomach contents | 1 microplastic | 3.3 mm | not available by species | not available by species | ATR-FTIR (subsample) | Andrade et al. 2019 |
| *Mugil cephalus* Linnaeus 1758 | Gut contents | 0 microplastics |  |  |  |  | Phillips and Bonner, 2015 |
| *Mugil curema* Valenciennes 1836 | Gut contents | 0-0.01 items/fish | < 5 mm |  | not available by species | stereomicroscope | Vendel et al. 2017 |
| *Neogobius melanostomus* (Pallas, 1814) | Gastrointestinal contents | 20 items/fish (data extrapolated from histogram) | < 5 mm |  |  | FTIR (subsample) | McNeish et al. 2018 |
| *Notemigonus crysoleucas* (Mitchill 1814) | Gastrointestinal contents | 0 microplastics |  |  |  |  | Phillips and Bonner, 2015 |
| *Noturus gyrinus* (Mitchill 1817) | Gut contents | microplastics present | not available by species |  | not available by species | FTIR (subsample) | Phillips and Bonner, 2015 |
| *Notropis atherinoides* Rafinesque, 1818 | Gastrointestinal contents | 70% of occurence (data extrapolated from histogram) | < 5 mm |  |  | stereomicroscope | Campbell et al. 2017 |
|  | Gastrointestinal contents | 13 items/fish (data extrapolated from histogram) | < 5 mm |  |  | FTIR (subsample) | McNeish et al. 2018 |
| *Notropis amabilis* (Girard 1856) | Gut contents | microplastics present | not available by species |  | not available by species | FTIR (subsample) | Phillips and Bonner, 2015 |
| *Notropis hudsonius* (Clinton, 1824) | Gastrointestinal contents | 15 items/fish (data extrapolated from histogram) | < 5 mm |  |  | FTIR (subsample) | McNeish et al. 2018 |
| *Notropis sabinae* Jordan & Gilbert 1886 | Gut contents | microplastics present | not available by species |  | not available by species | FTIR (subsample) | Phillips and Bonner, 2015 |
| *Notropis stramineus* (Cope, 1865) | Gastrointestinal contents | 13 items/fish (data extrapolated from histogram) | < 5 mm |  |  | FTIR (subsample) | McNeish et al. 2018 |
|  | Gut contents | microplastics present | not available by species |  | not available by species | FTIR (subsample) | Phillips and Bonner, 2015 |
| *Notropis volucellus* (Cope 1865) | Gut contents | microplastics present | not available by species |  | not available by species | FTIR (subsample) | Phillips and Bonner, 2015 |
| *Odontesthes bonariensis* (Valenciennes, 1835) | Gut contents | not available by species | < 5 mm | not available by species | not available by species | stereomicroscope | Pazos et al. 2017 |
| *Oligosarcus oligolepis* (Steindachner, 1867) | Gut contents | not available by species | < 5 mm | not available by species | not available by species | stereomicroscope | Pazos et al. 2017 |
| *Opsopoeodus emiliae* Hay 1881 | Gut contents | 0 microplastics |  |  |  |  | Phillips and Bonner, 2015 |
| *Oreochromis aureus* (Steindachner 1864) | Gut contents | microplastics present | not available by species |  | not available by species | FTIR (subsample) | Phillips and Bonner, 2015 |
| *Oreochromis niloticus* (Linnaeus, 1758) | Gastrointestinal contents | 20% of occurrence | < 5 mm | not available by species |  | ATR-FTIR (subsample) | Biginagwa et al. 2015 |
|  | Gastrointestinal contents | 75.9% of occurrence  7.5 ± 4.9 items/fish (mean) | < 5 mm | not available by species | fibers (65.3%), films (25.6%), fragments (8.5%) | ATR-FTIR (subsample) | Khan et al. 2020 |
|  | Gastrointestinal contents | 77% of occurrence | < 5 mm | not available by species | not available by species | ATR-FTIR (subsample) | Merga et al. 2020 |
| *Osmerus eperlanus* (Linnaeus, 1758) | Gastrointestinal contents | 20% of occurrence | < 5 mm | not available by species |  | FTIR (subsample) | McGoran et al. 2017 |
| *Ossubtus xinguense* Jégu, 1992 | Stomach contents | 11 microplastics | 1.7-4.3 mm  2.7±0.9 mm | not available by species | not available by species | ATR-FTIR (subsample) | Andrade et al. 2019 |
| *Parapimelodus valenciennis* (Lütken, 1874) | Gut contents | not available by species | < 5 mm | not available by species | not available by species | stereomicroscope | Pazos et al. 2017 |
| *Pelteobagrus fulvidraco* (Richardson, 1846) | Gastrointestinal contents | 0.33±0.58 items/fish | 0.3-0.5 mm | PE | lines | Raman | Zhang et al. 2017 |
| *Pelteobagrus nitidus* (Sauvage & Dabry de Thiersant, 1874) | Gastrointestinal contents | 0 microplastics |  |  |  |  | Zhang et al. 2017 |
| *Pelteobagrus vachelli* (Richardson, 1846) | Gastrointestinal contents | 1±1.41 items/fish | 0.5-0.6 mm | PE | sheet | Raman | Zhang et al. 2017 |
| *Perca fluviatilis* Linnaeus, 1758 | Gut contents | 0 microplastics |  |  |  |  | Faure et al. 2015 |
|  | Gastrointestinal contents | 25% of occurrence (data extrapolated from histogram) | not available by species |  | not available by species | hot needle | Roch et al. 2019 |
| *Pimelodus maculatus* Lacepéde, 1803 | Gut contents | not available by species | < 5 mm | not available by species | not available by species | stereomicroscope | Pazos et al. 2017 |
| *Pimephales promelas* (Rafinesque, 1820) | Gastrointestinal contents | 50% of occurrence | < 5 mm |  |  | stereomicroscope | Campbell et al. 2017 |
|  | Gastrointestinal contents | 5 items/fish (data extrapolated from histogram) | < 5 mm |  |  | FTIR (subsample) | McNeish et al. 2018 |
|  | Gut contents | 0 microplastics |  |  |  |  | Phillips and Bonner, 2015 |
| *Pimephales vigilax* (Baird & Girard 1853) | Gut contents | microplastics present | not available by species |  | not available by species | FTIR (subsample) | Phillips and Bonner, 2015 |
| *Platichthys flesus* Linnaeus, 1758 | Gastrointestinal contents | 75% of occurrence | < 5 mm | not available by species |  | FTIR (subsample) | McGoran et al. 2017 |
| *Poecilia vivipara* Bloch & Schneider 1801 | Gut contents | 0.08-0.11 items/fish | < 5mm |  | not available by species | stereomicroscope | Vendel et al. 2017 |
| *Pomoxis annularis* Rafinesque 1818 | Gut contents | 0 microplastics |  |  |  |  | Phillips and Bonner, 2015 |
| *Pomoxis nigromaculatus* (Lesueur 1829) | Gut contents | 0 microplastics |  |  |  |  | Phillips and Bonner, 2015 |
| *Pristobrycon* cf. *scapularis* | Stomach contents | 3 microplastics | 1.4-4.6 mm  2.6± | not available by species | not available by species | ATR-FTIR (subsample) | Andrade et al. 2019 |
| *Pristobrycon eigenmanni* (Norman, 1929) | Stomach contents | 0 microplastics |  |  |  |  | Andrade et al. 2019 |
| *Prochilodus lineatus* (Valenciennes, 1836) | Gut contents | not available by species | < 5 mm | not available by species | not available by species | stereomicroscope | Pazos et al. 2017 |
| *Pseudobagrus ussuriensis* (Dybowski, 1872) | Gastrointestinal contents | 1 item/fish | 1.8 mm | nylon | fragment | Raman | Zhang et al. 2017 |
| *Pseudoplatystoma corruscans* (Spix & Agassiz, 1829) | Gut contents | not available by species | < 5 mm | not available by species | not available by species | stereomicroscope | Pazos et al. 2017 |
| *Pseudorasbora parva* (Temminck and Schlegel, 1846) | Gastrointestinal contents | 5.6±3.9 items/g  2.5±1.8 items/fish | < 5 mm | not available by species | fibers (70.7%)  fragments (6.9%) | micro-FTIR | Jabeen et al. 2017 |
| *Pygocentrus nattereri* Kner, 1858 | Stomach contents | 0 microplastics |  |  |  |  | Andrade et al. 2019 |
| *Rhinosardinia bahiensis* (Steindachner 1879) | Gut contents | 0.14-0.35 items/fish | < 5 mm |  | not available by species | stereomicroscope | Vendel et al. 2017 |
| *Rutilus rutilus* (Linnaeus, 1758) | Gut contents | 0 microplastics |  |  |  |  | Faure et al. 2012 |
|  | Gut contents | 0 microplastics |  |  |  |  | Faure et al. 2015 |
|  | Gastrointestinal contents | 33% of occurrence | < 5 mm | PE, PP, PE, synthetic dye | fibers (75%), fragments (22.7%), films (2.3%) | Raman | Horton et al. 2018 |
|  | Gastrointestinal contents | 20% of occurrence (data extrapolated from histogram) | not available by species |  | not available by species | hot needle | Roch et al. 2019 |
| *Salmo trutta* Linnaeus, 1758 | Gastrointestinal contents | not available by species | not available by species |  | not available by species | fluorescence microscope | Simmerman and Coleman Wasik, 2020 |
| *Salvellinus fontinalis* (Mitchill, 1814) | Gastrointestinal contents | not available by species | not available by species |  | not available by species | fluorescence microscope | Simmerman and Coleman Wasik, 2020 |
| *Sander lucioperca* (Linnaeus, 1758) | Gastrointestinal contents | 0 microplastics |  |  |  |  | Roch et al. 2019 |
| *Sciades herzbergii* (Bloch 1794) | Gut contents | 0-0.08 items/fish | < 5 mm |  |  | stereomicroscope | Vendel et al. 2017 |
| *Serrasalmus* cf. *altispinis* | Stomach contents | 0 microplastics |  |  |  |  | Andrade et al. 2019 |
| *Serrasalmus manueli* Lacepède, 1803 | Stomach contents | 0 microplastics |  |  |  |  | Andrade et al. 2019 |
| *Serrasalmus rhombeus* (Linnaeus, 1766) | Stomach contents | 0 microplastics |  |  |  |  | Andrade et al. 2019 |
| *Silurus asotus* Linnaeus, 1758 | Gastrointestinal contents, gills, muscle | gastointestine: 22 microplastics  gills: 15 microplastic  muscles: 0 microplastics | < 5 mm | not available by species | not available by species | FTIR | Park et al. 2020 |
| *Silurus glanis* Linnaeus, 1758 | Gastrointestinal contents | 0 microplastics |  |  |  |  | Roch et al. 2019 |
| *Sinibrama wui* (Rendahl, 1933) | Gastrointestinal contents | 0 microplastics |  |  |  | Raman | Zhang et al. 2017 |
| *Siniperca chuatsi* (Basilewsky, 1855) | Gastrointestinal contents | 0 microplastics |  |  |  | Raman | Zhang et al. 2017 |
| *Squalidus argentatus* (Sauvage & Dabry de Thiersant, 1874) | Gastrointestinal contents | 0 microplastics |  |  |  | Raman | Zhang et al. 2017 |
| *Squalius cephalus* Linnaeus, 1758 | Liver, muscle, stomach contents | liver: 5% of occurrence  muscle: 0 microplastics  stomach: 15% of occurrence | liver: 0.286 mm  stomach: 1.44 mm | liver: PE(75%), PS (15%)  stomach: PET (n=5), PP (n=2) | liver: fragments (100%)  stomach: fibers (63%), fragments (22%) (data extrapolated from histogram) | Raman | Collard et al. 2018 |
| *Tometes ancylorhynchus* Andrade, Jégu & Giarrizzo, 2016 | Stomach contents | 0 microplastics |  |  |  |  | Andrade et al. 2019 |
| *Tometes kranponhah* Andrade, Jégu & Giarrizzo, 2016 | Stomach contents | 0 microplastics |  |  |  |  | Andrade et al. 2019 |
