# Supplementary material for: Freshwater wild biota exposure to microplastics: A global perspective
Source: Ecol Evol. 2021 Jul 9;11(15):9904–16. doi: 10.1002/ece3.7844 (PMC8328441; doi:10.1002/ece3.7844)
Supplement: Supplementary file 5 — Appendix S5 [file ECE3-11-9904-s002.docx]

**Appendix 5.** Focus of the investigations on microplastics in amphibian species.

| **Species** | **Analised component** | **Results** | **Particle size** | **Polymer types** | **Morphology** | **Analytical method** | **Reference** |
| --- | --- | --- | --- | --- | --- | --- | --- |
| *Anaxyrus americanus* (Holbrook, 1836) | digestive tract of adults | 0 microplastics |  |  |  |  | Schessl et al. 2019 |
| *Bufo gargarizans* Cantor, 1842 | tadpoles digested | 0.17-1.89 items/tadpole, 2.44-56.88 items/g | < 5 mm | not available by species | not available by species | ATR-micro-FTIR or SEM-EDS | Hu et al. 2018 |
| *Lithobates clamitans* (Latreille, 1801) | digestive tract of adults | 0 microplastics |  |  |  |  | Schessl et al. 2019 |
| *Lithobates palustris* (LeConte, 1825) | digestive tract of adults | 0 microplastics |  |  |  |  | Schessl et al. 2019 |
| *Lithobates pipiens* (Schreber, 1782) | digestive tract of adults | 0 microplastics |  |  |  |  | Schessl et al. 2019 |
| *Lithobates septentrionalis* (Baird, 1854) | digestive tract of adults | 0 microplastics |  |  |  |  | Schessl et al. 2019 |
| *Microhyla ornata* (Dumeril and Bibron, 1841) | tadpoles digested | 0.53-2.60 items/tadpole, 35.21-157.88 items/g | < 5 mm | not available by species | not available by species | ATR-micro-FTIR or SEM-EDS | Hu et al. 2018 |
| *Pelophylax nigromaculatus* (Hallowell, 1861) | tadpoles digested | 1.27-1.80 items/tadpole, 3.01-4.46 items/g | < 5 mm | not available by species | not available by species | ATR-micro-FTIR or SEM-EDS | Hu et al. 2018 |
| *Pelophylax ridibundus* (Pallas, 1771) | tadpoles digested | 302.62 ± 159.05 items/g | < 2 mm | not available by species | fibers (63.6%), fragments (42.9%) | ATR-FTIR | Karaoğlu and Gül 2020 |
| *Rana limnochari* Wiegmann, 1834 | tadpoles digested | 0 microplastics |  |  |  |  | Hu et al. 2018 |
| *Rana macrocnemis* (Boulenger, 1885) | tadpoles digested | 306.69 ± 182.72 items/g | < 2 mm | not available by species | fibers (57.1%), fragments (36.4%) | ATR-FTIR | Karaoğlu and Gül 2020 |
| *Triturus carnifex* (Laurenti, 1768) | stomach contents of adults | 59 microplastics in over 200 individuals | < 5 mm | polyacrylic polymers, PE | fibers, splinters, fragments | ATR-FTIR (subsample) | Iannella et al. 2020 |
