# Supplementary material for: Freshwater wild biota exposure to microplastics: A global perspective
Source: Ecol Evol. 2021 Jul 9;11(15):9904–16. doi: 10.1002/ece3.7844 (PMC8328441; doi:10.1002/ece3.7844)
Supplement: Supplementary file 6 — Appendix S6 [file ECE3-11-9904-s005.docx]

**Appendix 6.** Focus of the investigations on microplastics in bird species.

| **Species** | **Analised component** | **Results** | **Particle size** | **Polymer types** | **Morphology** | **Analytical method** | **Reference** |
| --- | --- | --- | --- | --- | --- | --- | --- |
| *Anas acuta* Linnaeus, 1758 | Digestive tract | microplastics are included in results on plastics |  |  |  | stereomicroscope | Holland et al. 2016 |
| *Anas Americana* (Gmelin, 1789) | Digestive tract | microplastics are included in results on plastics |  |  |  | stereomicroscope | Holland et al. 2016 |
| *Anas platyrhynchos* Linnaeus, 1758 | Gut contents | not available by species | not available by species | not available by species | not available by species | ATR-FTIR | Faure et al. 2015 |
| *Ardea cinerea* Linnaeus, 1758 | Gut contents | not available by species | not available by species | not available by species | not available by species | ATR-FTIR | Faure et al. 2015 |
| *Branta canadensis* (Linnaeus, 1758) | Digestive tract | microplastics are included in results on plastics |  |  |  | stereomicroscope | Holland et al. 2016 |
| *Cinclus cinclus Linnaeus*, 1758 | Rigurgitates, faeces | 74.2% microplastics, 25.8% meso e macroplastics, 0.9 ± 0.1 items/sample, 11.3 ± 1.6 items/g dw; rigurgitates: 50% of occurrence, faeces: 45% of occurrence | microplastics 0.5-5 mm | polyvinyl alcohol (37.7%), PE (26.4%), several in minor percentages | fibres (>95%) | FTIR | D'Souza et al. 2020 |
| *Cygnus olor* (Gmelin, 1789) | Gut contents | not available by species | not available by species | not available by species | not available by species | ATR-FTIR | Faure et al. 2015 |
| *Larus delawarensis* Ord, 1815 | Gastrointestinal contents | microplastics are included in results on plastics |  |  |  | FTIR or Raman | Thaysen et al. 2020 |
| *Phalacrocorax auritus* (Lesson, 1831) | Stomach contents of chicks | 86% occurrence of antropogenic debris (not clear distinction of microplastics in results) |  |  | mainly fibres | FTIR or Raman | Brookson et al. 2019 |
| *Podiceps nigricollis* Brehm, 1831 | not specified | 0 microplastics |  |  |  |  | Faure et al. 2012 |
| Scientific names of 15 bird species not included | Digestive tract | microplastics are included in results on plastics |  |  |  | stereomicroscope | Holland et al. 2016 |
